# A shift from inorganic to organic nitrogen-dominance shapes soil microbiome composition and co-occurrence networks

**DOI:** 10.3389/fmicb.2022.1074064

**Published:** 2022-12-19

**Authors:** Yue Xin, Yu Shi, Wei-Ming He

**Affiliations:** ^1^College of Forestry, Hebei Agricultural University, Baoding, China; ^2^State Key Laboratory of Crop Stress Adaptation and Improvement, School of Life Sciences, Henan University, Kaifeng, China

**Keywords:** α diversity, β diversity, microbial networks, plant functional traits, soil abiotic properties

## Abstract

Soil microbiomes are characterized by their composition and networks, which are linked to soil nitrogen (N) availability. In nature, inorganic N dominates at one end and organic N dominates at the other end along soil N gradients; however, little is known about how this shift influences soil microbiome composition and co-occurrence networks, as well as their controls. To this end, we conducted an experiment with the host plant *Solidago canadensis*, which was subject to three N regimes: inorganic N-dominated, co-dominated by inorganic and organic N (CIO), and organic N-dominated. Organic N dominance exhibited stronger effects on the composition and co-occurrence networks of soil microbiomes than inorganic N dominance. The predominant control was plant traits for bacterial and fungal richness, and soil pH for keystone species. Relative to the CIO regime, inorganic N dominance did not affect fungal richness and increased keystone species; organic N dominance decreased fungal richness and keystone species. Pathogenic fungi and arbuscular mycorrhizal fungi were suppressed by organic N dominance but not by inorganic N dominance. These findings suggest that the shift from soil inorganic N-dominance to soil organic N-dominance could strongly shape soil microbiome composition and co-occurrence networks by altering species diversity and topological properties.

## Introduction

In general, soils harbor the majority of microbes, and soil microbiomes have importantly ecological functioning (Berendsen et al., 2012; Bardgett and van der Putten, 2014; Wall et al., 2015; Pérez-Valera et al., 2020). As early as the early 20^th^ century, Beijerinck and Becking pointed out “Everything is everywhere, but the environment selects” (*cf.* Tate, 2021). Since then this prevailing paradigm has been directing microbial studies. Essentially, environmental selections are derived from two categories of driving forces: changes in resources (e.g., soil water and nutrients) and conditions (e.g., soil acidity; Tate, 2021). Thus, a fundamental theme in microbial research is to understand how environmental selections shape the composition, structure, and function of soil microbiomes (Tate, 2021).

Of all environmental selections, soil nitrogen (N) is one of the key resource selectors underlying the composition and co-occurrence networks of soil microbiomes because soil N availability has profound effects on soil microbiomes *via* direct and indirect pathways (Yeoh et al., 2016; Marupakula et al., 2017; Wang et al., 2019; Zhao et al., 2020; Hu et al., 2022). Related mechanisms mainly encompass the following aspects: altering soil pH, competition for N between microbes and plants, and plant–soil feedbacks (Kuzyakov and Xu, 2013; Fierer, 2017; Tate, 2021). Importantly, human activities are strongly altering soil microbiomes by changing soil N availability (Zhang et al., 2018).

Although the importance of soil N has been recognized in shaping soil microbes, previous studies have largely focused on soil inorganic N but not soil organic N (Yeoh et al., 2016; Marupakula et al., 2017; Zhao et al., 2020). Specifically, these studies concentrate on the dose effects of different inorganic N forms. Indeed, soil inorganic N and soil organic N both take part in the above-mentioned mechanisms (Kuzyakov and Xu, 2013), and they substantially shift along environmental gradients (Yu and He, 2021, 2022). Additionally, human activities (e.g., fertilization and fossil combustion) could rapidly alter the relative dominance of soil inorganic and organic N. Taken together, there is a need to understand how the shift from soil inorganic N dominance to soil organic N dominance shapes the composition and co-occurrence networks of soil microbiomes under more realistic scenarios.

While the awe-inspiring size of soil microbial research has been performed, we are still faced with questions about how to best sample microbial communities to maximize what we can learn about how they are structured and how they change (Jansson and Prosser, 2013; Hugerth and Anderson, 2017). For example, a lack of clear backgrounds regarding microbial habitats (e.g., host plants and soil conditions) hampers to identify the factors that shape soil microbial communities (Chen et al., 2019; Revillini et al., 2019). One way to tackle this problem is to select similar or even identical target systems to normalize their background. Based on this idea, we set up a study system with the same plant communities and soil biotic and abiotic properties at the beginning of our research (see the Materials and Methods for more details). We then introduced experimental manipulations (i.e., altering soil N dominance by adding different proportions of inorganic N and organic N) after a few months to address how the change in soil N dominance influences the composition and co-occurrence networks of soil microbiomes.

Here we focused exclusively on soil bacteria and fungi because both are prime components of soil microbes (Tate, 2021). Fauci and Dick (1994) detected that organic N enhanced soil microbial activities while inorganic N suppressed microbial activities; in contrast, Zhang et al. (2021) observed that inorganic N had stronger impacts on soil bacterial communities than organic N. Our previous studies found that organic N had stronger facilitation on the growth of *Solidago canadensis* than inorganic N (Yu et al., 2016; Yu and He, 2021, 2022). These findings suggest that inorganic N and organic N could differentially alter the root exudates of *S. canadensis* and yield different impacts on soil microbes (Sun and He, 2019; Yu et al., 2022). Based on the current knowledge, we proposed the following hypotheses. (1) Organic N dominance could have stronger effects on the composition and co-occurrence networks of soil microbiomes than inorganic N dominance, and (2) plant functional traits might play a more important role than soil abiotic properties in shaping soil microbiomes along the soil N dominance gradient. To test these hypotheses, we carried out a 3-year experiment and determined a suite of leaf and whole-plant traits and soil biotic and abiotic properties.

## Materials and methods

### A 3-year field experiment

The experiment was implemented in spring 2017 at the China National Botanical Garden (39.98 °N, 116.20 °E, and 80 m above sea level), where the mean annual precipitation is 500 mm and the mean annual air temperature is 12°C (He and Sun, 2016). We selected *Solidago canadensis* L. as a host plant species because it is a widespread forb in China; its seeds were field-collected from a wild population (Jiujiang, Jiangxi Province) so that they were genetically similar. This experiment focused exclusively on the relative dominance of soil inorganic and organic N for the following reasons. Soil organic N is exceptionally more available than soil inorganic N, and importantly soil organic N can act as an important N source available to plants (Lipson and Näsholm, 2001; Warren, 2014; Yu and He, 2021, 2022); to date, limited studies have addressed how soil organic N influences soil microbiomes, although its importance in soil ecology has long been recognized (Lipson and Näsholm, 2001; Warren, 2014; Zhang et al., 2021; Rozmoš et al., 2022).

Prior to our experiment, 4.5-L sand, 4.5-L vermiculite, and 1.0-L native topsoil (cinnamon soil) were mixed thoroughly and then a container (40 cm length × 30 cm width × 15 cm depth) was filled by the mixture. Sand and vermiculite were purchased and the native soil was collected from the experimental site. The seeds of *S. canadensis* were planted in the above containers; once seeds germinated, seedlings were gradually thinned to identical individuals per container. We set up a soil N dominance gradient because environmental gradients are more useful for understanding reaction norms compared to two states of a given factor (Hulme, 2007). In the field, plant-available N substantially varies with habitat: inorganic N (i.e., nitrate and ammonium) dominates at one end of the N availability gradient and organic N (i.e., soil free amino acids) dominates at the other end. Based on our field survey, we set up three N regimes: inorganic N-dominated (3:1 inorganic to organic N, INO), co-dominated by inorganic and organic N (2:2 inorganic to organic N, CIO), and organic N-dominated (1:3 inorganic to organic N, ORG). Inorganic N included NH_4_Cl and NaNO_3_, while organic N included 15 amino acids (i.e., alanine, arginine, aspartic acid, glutamic acid, glycine, histidine, isoleucine, leucine, lysine, methionine, phenylalanine, proline, serine, tyrosine, and valine), which commonly appear in soil solutions (Yu and He, 2021, 2022). Note that amino acids are also a carbon source for microbes (see Discussion for more details). We based the N level on field N availability; 830 mg of N per year was supplied to each container, and each N form was supplied on the basis of equivalent N availability. We also provided an N-free Hoagland solution to each container during the experiment. Each N regime was initially replicated 20 times so that there were 60 experimental containers in total (3 N regimes × 20 replicates per N regime).

### Determination of soil abiotic properties and plant traits

In August 2020, we determined soil pH, nitrate (NO_3_^−^-N), and ammonium (NH_4_^+^-N). Soil pH was measured with a Sartorius PB-10 pH meter. Briefly, approximately 50 g of soil was collected from growth containers and air dried, 5 g of air-dried soil was sieved with a 2-mm mesh and then dissolved in 12.5-ml distilled water, and soil solutions were measured with the above pH meter. NO_3_^−^-N and NH_4_^+^-N were determined with a continuous flow analyzer (AA3, Bran and Luebbe, Norderstedt, Germany). Briefly, soil samples were sieved with a 2-mm mesh, 10 g of sieved soil was dissolved in 50-ml KCl solution for 1 h, and the filtrate was determined with the above flow analyzer.

We determined leaf functional traits (i.e., leaf C, leaf N, and leaf C/N) and whole-plant traits (i.e., plant height and aboveground biomass). Leaf C (mg g^−1^) and leaf N (mg g^−1^) were determined using an elemental analyzer as previously described by Wang et al. (2021). At the peak growth (i.e., August 2020), we first determined the height of individual plants with a ruler and then harvested shoots per container for aboveground biomass. More specifically, shoots were harvested and then oven-dried at 65°C for 48 h.

### Determination of soil microbes

In August 2020, five topsoil samples were taken from a container, thoroughly mixed as a composite sample, and sieved with a 2-mm mesh to exclude debris and roots. All the composite samples were immediately sent to the laboratory for microbial DNA sequencing. We used the Magnetic Soil and Stool DNA Kit (Tiangen Biotech, Beijing, China) to extract soil microbial DNA according to the manufacturer’s protocols. The purified DNA was assessed with a NanoDrop 2000 UV-VIS spectrophotometer and was quality-checked with 1% agarose gel electrophoresis. The 16S rRNA V4 region was amplified for soil bacteria with two universal primers 505F and 806R (Zhou et al., 2016), and the ITS1 genes were amplified for soil fungi with primer pairs of ITS1F and ITS2R (Gardes and Bruns, 1993). The polymerase chain reactions (PCRs) were performed in 30 μl reactions with 15 μl of Phusion^®^ High-Fidelity PCR Master Mix (New England Biolabs). Briefly, the following protocols were used for PCRs: denaturation at 98°C for 1 min, followed by 30 cycles of denaturation at 98°C for 10 s, annealing at 50°C for 30 s, elongation at 72°C for 30 s, and ending with 72°C for 5 min.

QIIME2 software (qiime2-2022.2; Bolyen et al., 2019) was used to process the raw FASTQ data. Paired-end sequence reads were quality filtered, denoised, chimera removed, and generated into amplicon sequence variants (ASVs) using the dada2 plugin (Callahan et al., 2016). The forward and reverse reads were trimmed at 210 and 230 bp for bacteria, and at 220 and 220 bp for fungi to remove low-quality regions from the sequences. The q2-feature-classifier plugin and the Naïve Bayes classifier trained on the SILVA 138 and UNITE Fungal ITS reference sequences were used for taxonomic classification of bacteria and fungi. Sequences were rarefied at the lowest number per sample after non-bacterial and non-fungal sequences were removed, generating a data set with a total of 16,807 ASVs for bacteria and 4,380 ASVs for fungi. To obtain a comprehensive overview of microbial diversity within and between samples, the observed ASV richness and Bray-Curtis distance matrices were calculated for bacteria and fungi. The fungal ASVs were further categorized into pathogenic fungi, saprotrophic fungi, and arbuscular mycorrhizal fungi using the expert-curated database FUNGuild (Nguyen et al., 2016). Note that only those species with a “probable“or “highly probable” confidence ranking for the guild assignment were used when analyzing fungal functional guilds (Lekberg et al., 2021).

### Data analysis

Co-occurrence networks of soil microbes were established on the iNAP platform^1^ by Feng et al. (2022). To increase the reliability of each network, the ASVs, with relative abundances of <0.01% and occurred in <70% of the samples from each N regime, were removed before network analysis. Thus, 626 bacterial ASVs and 139 fungal ASVs under the INO regime, 644 bacterial ASVs and 155 fungal ASVs under the CIO regime, and 505 bacterial ASVs and 114 fungal ASVs under the ORG regime were retained, combined, and used to create bacterial-fungal co-occurrence networks. The SparCC analysis (Friedman and Alm, 2012) was used to determine the pair-wise relationships among the ASVs, and the correlations between-0.8 and 0.8 with *p* > 0.05 were removed. To unveil the differences in network properties among N regimes, we analyzed topological properties such as average degree, average clustering coefficient, and modularity. Two parameters, within-module degree (*Zi*) and among-module connectivity (*Pi*), were further used to characterize the topological roles of different ASVs. The nodes in each network were categorized into (i) network hubs (*Zi* > 2.5 and *Pi* > 0.62, highly connected nodes within and among modules), (ii) module hubs (*Zi* > 2.5, highly connected nodes within a module), (iii) connectors (*Pi* > 0.62, highly connected nodes among modules), and (iv) peripherals (*Zi* < 2.5 and *Pi* < 0.62, nodes with few links or unconnected with other nodes). Keystone species are defined as nodes that act as connectors or hubs in a network. The networks were visualized with Gephi 0.9.2.

One-way analysis of variance with LSD *post-hoc* tests was employed to examine the differences in soil abiotic properties (pH, ammonium, and nitrate), leaf functional traits (C, N, and C/N), and whole-plant traits (plant height and aboveground biomass) among three N regimes. The soil ammonium concentration was log transformed to meet the assumption of normality. To test the impacts of soil N regimes on microbial richness, generalized linear models with a Poisson distribution were performed. We further selected the best predictors for microbial richness under each N regime using the bioenv method. To quantify the relative importance of explanatory variables in shaping microbial richness, the relative contribution of each explanatory variable was assessed using the hierarchical partitioning method.

For microbial composition, Kruskal-Wallis tests were implemented to assess the impacts of N regimes on the relative abundance of top 10 bacterial phyla and 10 fungal classes, as well as three fungal functional guilds. Principal coordinate analysis (PCoA) based on Bray–Curtis distance matrices was used to depict the variation of bacterial and fungal diversities, followed by a permutational multivariate analysis of variance (PERMANOVA), a multiple-response permutation procedure (MRPP), and an analysis of similarities (ANOSIM) to test the significance of N regimes in microbial community composition. Venn analysis was performed to distinguish the number of unique and shared ASVs among three N regimes. Partial Mantel tests were used to quantify the correlations of explanatory variables (i.e., soil abiotic properties and plant traits) with bacteria, fungi, and keystone species. All statistical analyses were performed using the R software (version 4.2.1) with the packages of agricolae (version 1.3–5; Mendiburu, 2021), car (version 3.1.1; Fox and Weisberg, 2019), hier.part (version 1.0.6; Nally and Walsh, 2004), MASS (version 7.3.57; Venables and Ripley, 2002), and vegan (version 2.6–2; Oksanen et al., 2022).

## Results

### Soil abiotic properties and plant traits along a soil N gradient

Overall, the shift in the relative dominance of soil inorganic and organic N altered soil abiotic properties and plant traits (Table 1). Compared to the CIO regime, the INO and ORG regime decreased soil pH; the INO regime failed to influence ammonium and the ORG regime increased ammonium (Table 1). Changing soil N dominance had no effects on nitrate and plant aboveground biomass (hereafter biomass; Table 1). Plant height (hereafter height), leaf C, leaf N, and C/N significantly shifted along the soil N dominance gradient (Table 1). For example, height increased and leaf C decreased at the INO and ORG end relative to the CIO regime.

**Table 1:** Soil abiotic properties and plant traits under three different N regimes.

	INO	CIO		ORG
Soil pH	7.44 ± 0.08 b	7.69 ± 0.11 a		7.35 ± 0.09 b
NH_4_^+^-N (mg kg^−1^)	2.75 ± 0.21 b	2.69 ± 0.25 b		4.06 ± 0.52 a
NO_3_^−^-N (mg kg^−1^)	11.48 ± 1.82 a	12.87 ± 2.32 a		15.76 ± 2.04 a
Aboveground biomass (g)	146.56 ± 34.34 a	85.1 ± 16.98 a		91.59 ± 11.36 a
Plant height (cm)	135.15 ± 11.87 a	115.45 ± 6.97 b		116.26 ± 4.76 ab
Leaf carbon (mg g^−1^)	44.52 ± 0.12 b	44.90 ± 0.09 a		44.25 ± 0.11 c
Leaf nitrogen (mg g^−1^)	3.65 ± 0.02 a	3.55 ± 0.04 ab		3.52 ± 0.04 b
Leaf carbon/nitrogen	12.21 ± 0.05 b	12.67 ± 0.14 a		12.58 ± 0.14 a

INO, inorganic N-dominated; CIO, co-dominated by inorganic and organic N; ORG, organic N-dominated. Different lowercase letters within a row indicate significant differences among regimes at *p* < 0.05.

### Composition of soil microbiomes along a soil N gradient

**Figure 1:**
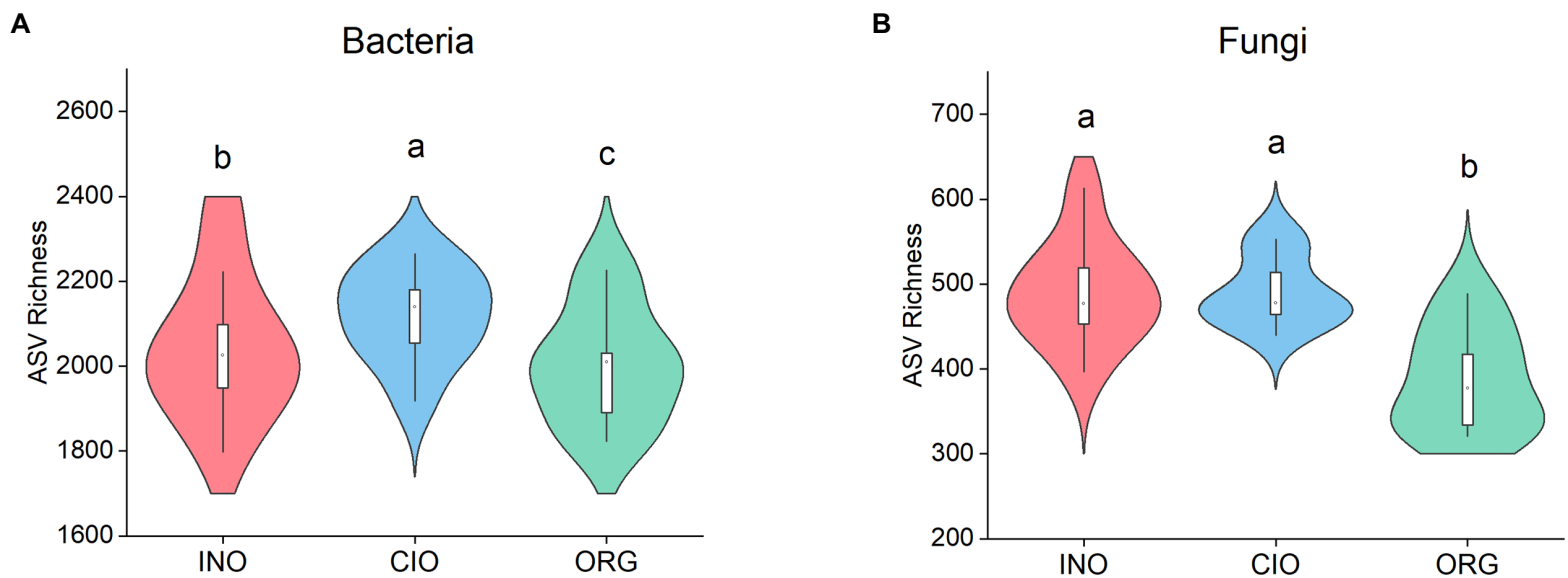
Soil bacterial richness **(A)** and soil fungal richness **(B)** under three soil N regimes. INO, inorganic N-dominated; CIO, co-dominated by inorganic and organic N; ORG, organic N-dominated. Different lowcase letters indicate significant differences at *p* < 0.05.

Across all soil samples, 1,605,405 high-quality bacterial sequence reads with 16,807 ASVs and 2,081,627 high-quality fungal sequence reads with 4,380 ASVs were obtained. The bacterial richness was highest under the CIO regime, followed by the INO and ORG regime (Figure 1A). The fungal richness was greater under the INO and CIO regime than the ORG regime (Figure 1B). For bacterial richness, the best predictors were ammonium and biomass under the INO and ORG regime, and leaf carbon under the CIO regime (Table 2). For fungal richness, the best predictors were soil pH and height under the INO regime, biomass and leaf carbon under the CIO regime, and ammonium and height under the ORG regime (Table 2). In terms of relative contributions, the predominant contributor to bacterial richness was biomass under the INO and ORG regime, and leaf carbon under the CIO regime; the predominant contributor to fungal richness was soil pH under the INO regime, biomass and leaf carbon under the CIO regime, and height under the ORG regime (Table 2).

**Table 2:** The best predictors and relative contribution to soil bacterial and fungal richness.

Models	N regime	Predictor	Correlation	*R* ^2^	Relative contribution (%)
Bacteria	INO	NH_4_^+^-N	0.56	0.69	2
		Biomass			98
	CIO	Leaf carbon	0.31	0.18	100
	ORG	NH_4_^+^-N	0.36	0.24	7
		Biomass			93
Fungi	INO	pH	0.63	0.70	97
		Height			3
	CIO	Biomass	0.72	0.80	51
		L carbon			49
	ORG	NH_4_^+^-N	0.20	0.38	30
		Height			70

**Figure 2:**
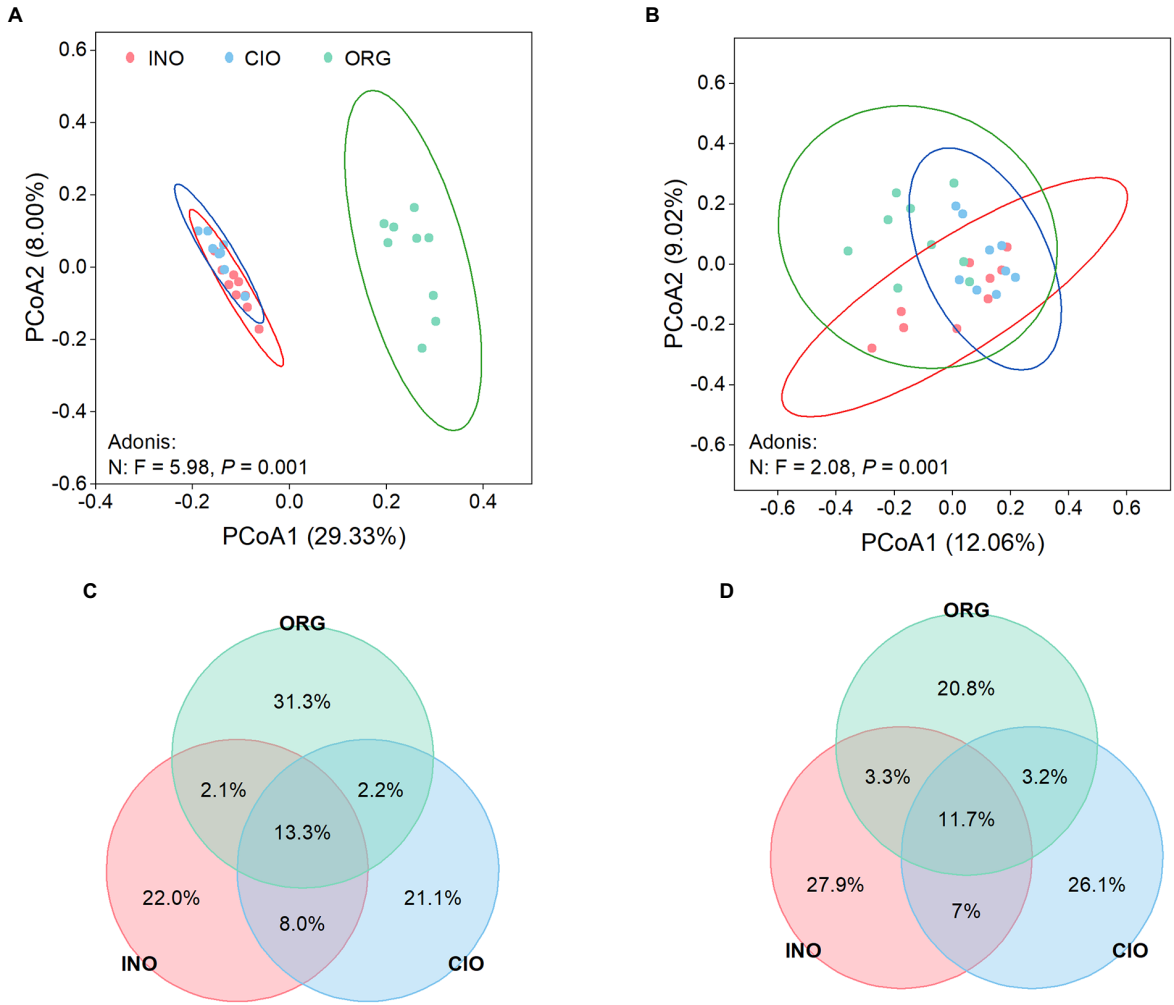
Principal coordinate analysis of soil bacteria **(A)** and soil fungi **(B)** under three soil N regimes. Venn diagrams depict the percentage of shared and unique ASVs of soil bacteria **(C)** and soil fungi **(D)** among three soil N regimes. INO, inorganic N-dominated; CIO, co-dominated by inorganic and organic N; ORG, organic N-dominated.

The PCoA ordination and dissimilarity analysis revealed that the composition of bacterial and fungal communities was separated distinctly along the soil N dominance gradient: that is, the shift in soil N dominance led to microbial differentiation (Figures 2A,B; Supplementary Table S1). Venn plots strongly indicated that both bacterial and fungal communities had a greater proportion of unique ASVs under each N regime, thereby suggesting a strong variation of microbial communities along the soil N dominance gradient (Figures 2C,D).

**Figure 3:**
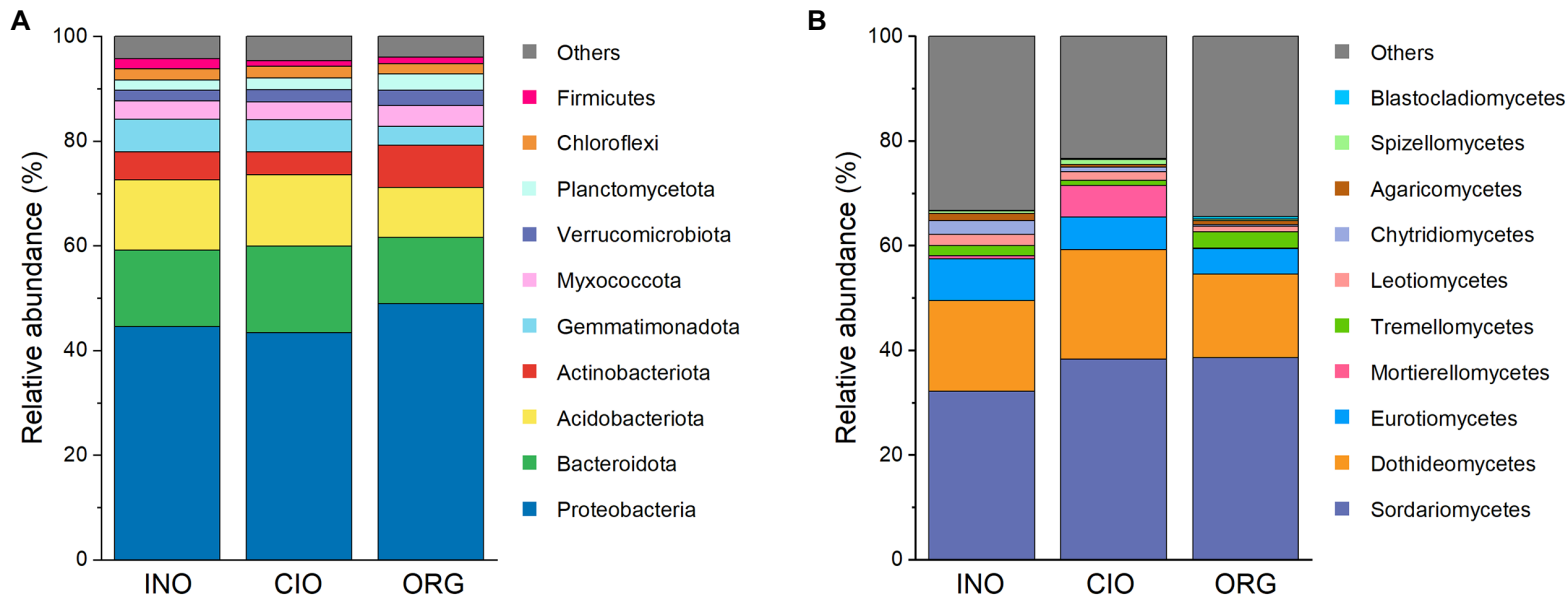
Relative abundance of phylum-level bacteria **(A)** and class-level fungi **(B)** under three soil N regimes. INO, inorganic N-dominated; CIO, co-dominated by inorganic and organic N; ORG, organic N-dominated.

In terms of the top phyla/classes, there were distinct differences in their relative abundance among three N regimes, regardless of bacteria (Figure 3A; Supplementary Table S2) or fungi (Figure 3B; Supplementary Table S2). The top 10 phyla accounted for 96% of the total bacterial abundance (Figure 3A). Proteobacteria, Actinobacteriota, Myxococcota, and Verrucomicrobiota were more abundant under the ORG regime than the INO or CIO regime, while the opposite was true for Firmicutes (Supplementary Table S2). In contrast, Bacteroidota were most abundant under the CIO regime (Supplementary Table S2). Fungal communities were overwhelmingly dominated by Sordariomycetes (36%), and the subordinates included Dothideomycetes (18%), Eurotiomycetes (6%), Mortierellomycetes (2%), and Tremellomycetes (2%; Figure 3B). Relative to the CIO regime, the ORG regime decreased the abundance of Mortierellomycetes and Spizellomycetales, and increased Tremellomycetes abundance (Supplementary Table S2). Moreover, the Blastocladiomycete abundance increased with organic N dominance (Supplementary Table S2).

**Figure 4:**
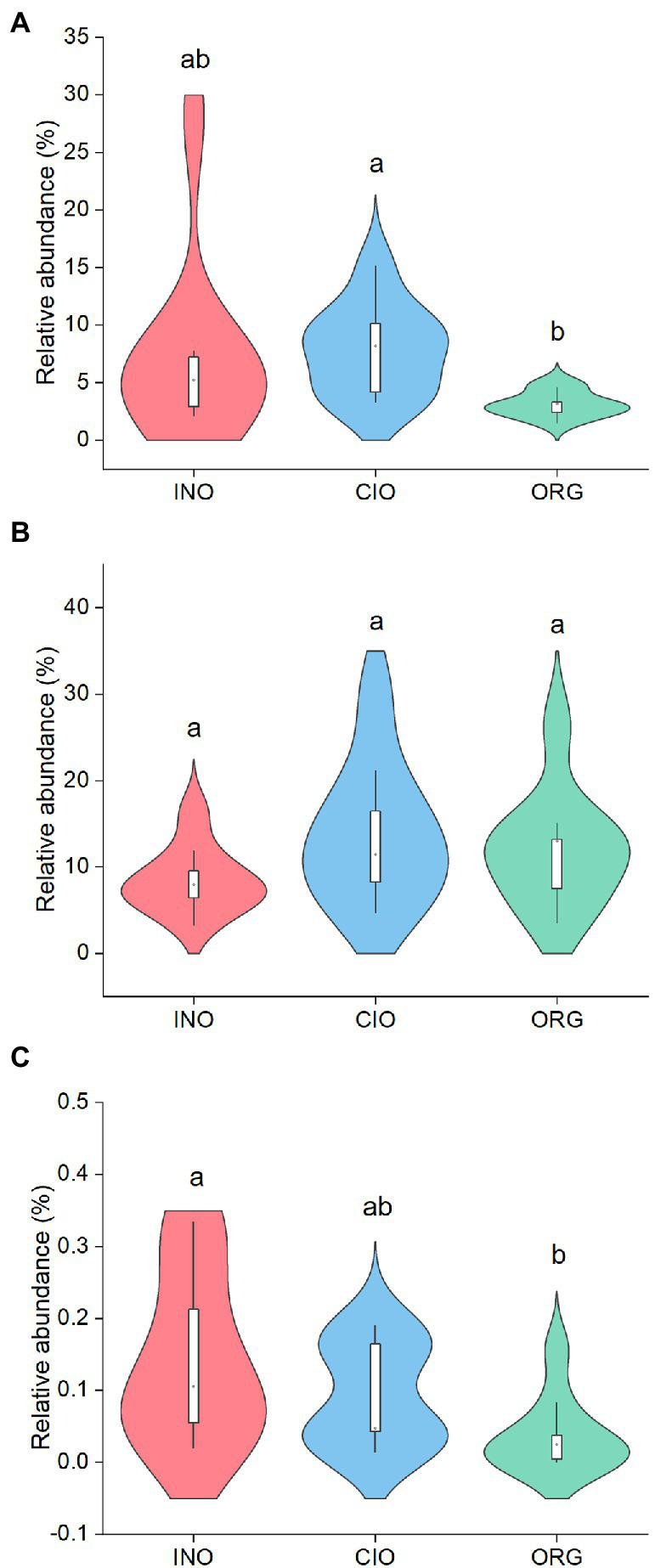
Relative abundance of pathogenic fungi **(A)**, saprotrophic fungi **(B)**, and arbuscular mycorrhizal fungi **(C)** under three soil N regimes. INO, inorganic N-dominated; CIO, co-dominated by inorganic and organic N; ORG, organic N-dominated. Different lowcase letters indicate significant differences at *p* < 0.05.

Based on the FUNGuild dataset, 31% of the fungal ASVs were identified as known guilds, and 78% of those ASVs had a “highly probable” or “probable” confidence ranking. The N regimes had a variety of effects on fungal functional guilds (Figure 4). Specifically, pathogenic fungi were most abundant under the CIO regime, followed by the INO and ORG regime (Figure 4A); saprotrophic fungi were similar among three N regimes (Figure 4B); arbuscular mycorrhizal fungi decreased with organic N dominance (Figure 4C).

### Co-occurrence networks of soil microbiomes along a soil N gradient

**Figure 5:**
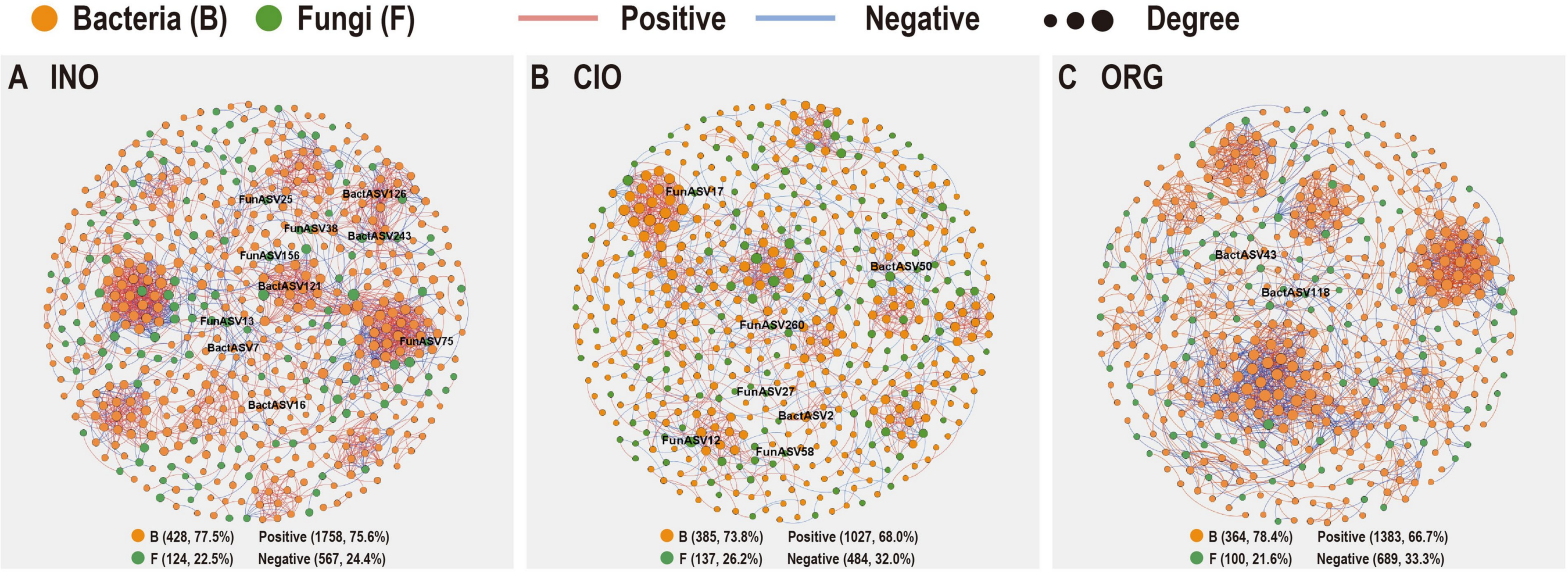
Co-occurrence network patterns of soil bacterial and fungal communities under the INO regime **(A)**, CIO regime **(B)**, and ORG regime **(C)**. INO, inorganic N-dominated; CIO, co-dominated by inorganic and organic N; ORG, organic N-dominated.

The INO network (552 nodes and 2,325 edges) and ORG network (464 nodes and 2072 edges) had larger average degrees and average clustering coefficients than the CIO network (522 nodes and 1,151 edges), suggesting that the increase in soil inorganic or organic N could strongly enhance the complexity of networks (Figure 5; Table 3). Soil relative N dominance also strongly influenced the keystone species of bacteria-fungi networks (Supplementary Table S3). Overall, the number of keystone species decreased with organic N dominance (Figure 5; Supplementary Table S3). Specifically, there were eight connectors and two module hubs in the INO network, four connectors and three module hubs in the CIO network, and two connectors in the ORG network (Supplementary Table S3). Connectors included phylotypes from Sordariomycetes, Alphaproteobacteria, and Gammaproteobacteria in the INO network, Eurotiomycetes and Dothideomycetes in the CIO network, and Alphaproteobacteria and Gammaproteobacteria in the ORG network (Supplementary Table S3). The module hubs were primarily classified into Alphaproteobacteria, Gammaproteobacteria, and Holophagae. Importantly, specific keystone species differed among three N regimes; an increase in keystone species under the INO regime was achieved by increasing bacterial keystone species, and a decrease in keystone species under the ORG regime was achieved by decreasing bacterial and fungal keystone species at the same (Supplementary Table S3).

**Table 3:** Topological properties of bacteria-fungi networks under three N regimes.

	INO	CIO	ORG
Node	552	522	464
Edge	2,325	1,151	2072
Complexity	4.21	2.21	4.41
Average degree	8.42	5.79	8.93
Average clustering coefficient	0.46	0.39	0.49
Transitivity	0.72	0.65	0.67
Positive correlations (%)	75.6	68	66.7
Negative correlations (%)	24.4	32	33.3
Modularity	0.812	0.831	0.759

INO, inorganic N-dominated; CIO, co-dominated by inorganic and organic N; ORG, organic N-dominated.

### Associations of soil microbiomes with soil abiotic properties and plant traits

**Figure 6:**
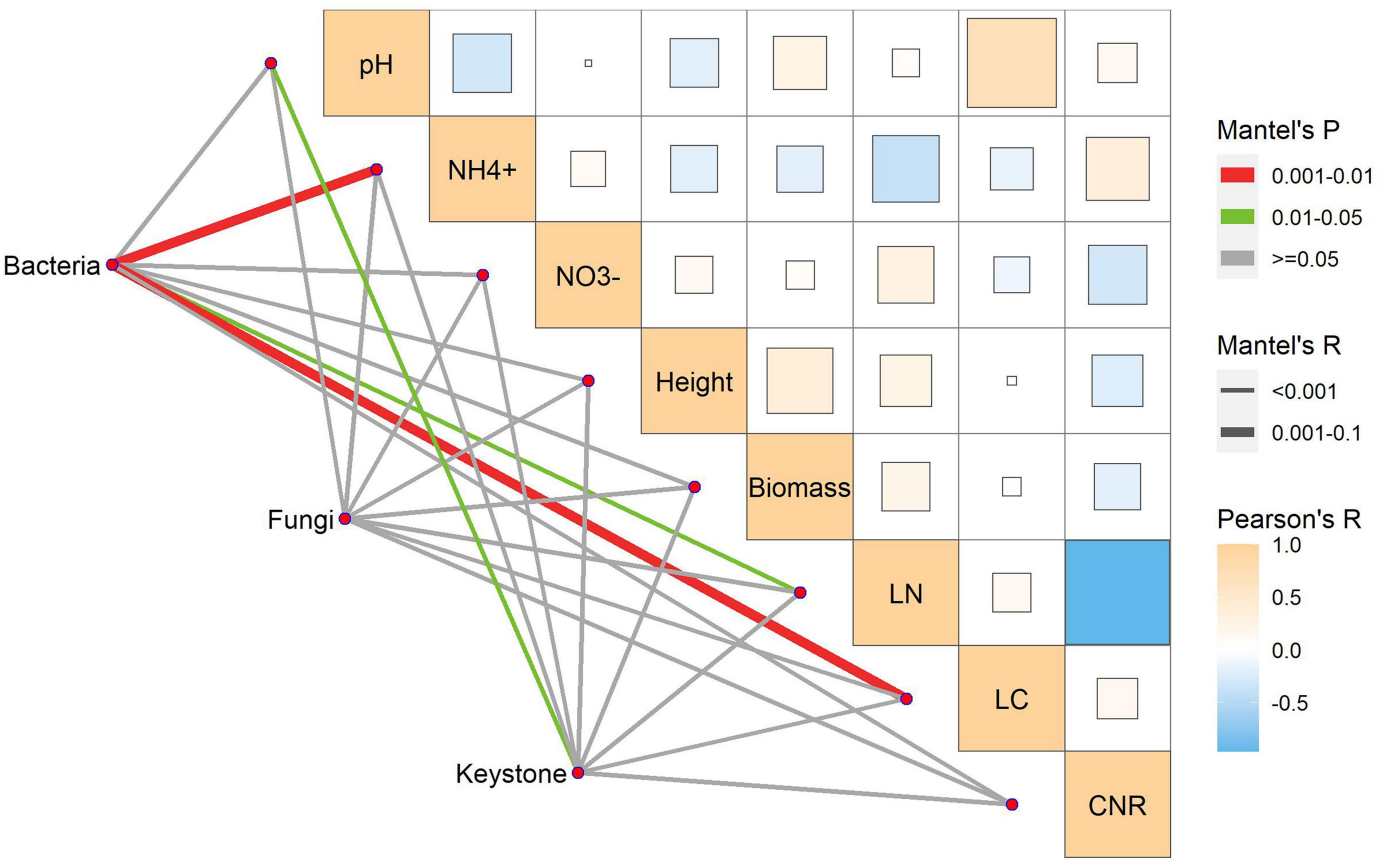
Mantel test for the associations of bacteria, fungi, and keystone species with soil abiotic properties and plant traits.

We used the Mantel test to identify the factors that shaped soil microbes and their keystone species. Soil abiotic properties and plant functional traits differentially shaped soil bacteria, fungi, and keystone species (Figure 6). In terms of controls, bacterial communities were significantly correlated with soil ammonium, leaf C, and leaf N; fungal communities were not significantly correlated with all the factors; keystone species were significantly correlated with soil pH rather than the other factors (Figure 6). Consequently, the dominant determinants of the soil microbiome varied with microbial identities.

## Discussion

Overall, amino acid N dominance exhibited stronger effects on the composition and co-occurrence networks of the soil microbiome than inorganic N dominance. This finding strongly supports our first hypothesis and the leading notion that N addition can shape soil microbes (Yeoh et al., 2016; Marupakula et al., 2017; Zhao et al., 2020). We proposed a couple of possibilities for this result. First, in our experiment, soil phosphorus was enough for *S. canadensis* individuals on the basis of our field survey (Yu et al., 2016), and *S. canadensis* individuals can allocate more biomass to roots when soil phosphorus is adequate (Yu et al., 2016). Second, there is usually a positive correlation between root biomass and its exudates (Sun and He, 2019; Yu et al., 2022). In other words, increased root biomass favors the production of root exudates, which play a key role in mediating soil microbes (Sun and He, 2019; Yu et al., 2022). Third, soil abiotic properties had stronger responses at the amino acid N-dominant end than at the inorganic N-dominant end, and efficient exoenzymes could be lacking in the presence of organic N (Rozmoš et al., 2022). These differences might exhibit contrasting effects on soil microbes.

We observed that plant functional traits did not consistently exhibit greater impacts on the composition and co-occurrence networks of the soil microbiome than soil abiotic properties and that their contributions to soil microbiomes varied with the metrics of interest. For example, the predominant controls were plant traits for microbial richness, and soil pH for keystone species. Thus, these findings partly support our second hypothesis and also provide new evidence for the complexity and diversity of predominant controls for soil microbes (Fauci and Dick, 1994; Chen et al., 2019; Tate, 2021). Our study system had the same host plant species and soil initially so that the differences in soil abiotic properties and plant functional traits could be ascribed primarily to N treatments. The likely mechanisms underlying these predominant controls at least include soil pH change, interspecific associations, plant–soil feedbacks, and the responsiveness of study entities (Kuzyakov and Xu, 2013; Fierer, 2017; Tate, 2021).

We found that the composition of soil microbiomes was varied along the soil N dominance gradient, similar to some previous studies (Marupakula et al., 2017; Wang et al., 2019; Zhao et al., 2020). Overall, the main driver of microbial richness was whole-plant traits (i.e., aboveground biomass and plant height) but not soil abiotic properties. Thus, plant growth might play a key role in shaping soil microbes *via* root exudates (Sun and He, 2019; Yu et al., 2022). Our molecular data showed that photosynthetic pathways were upregulated at the inorganic N-dominated end and N metabolism pathways were upregulated at the amino acid N-dominated end (data not shown). Therefore, plants exhibited contrasting N use strategies under different N regimes and these strategies favor to enhance plant growth. Our data also imply that the shift from inorganic N-dominance to amino acid N-dominance could strongly alter the beta diversity of soil bacteria and fungi, which could be linked to rapid species turnover driven by N availability (Yu and He, 2019).

In our study, soil bacteria and fungi had contrasting sensitivity to soil N dominance. Such a phenomenon has been detected in a previous study (Marupakula et al., 2017). An increase in inorganic N and amino acid N decreased bacterial richness but this decrease was much stronger under the amino acid N-dominant regime than under the inorganic N-dominant regime; the predominant control for bacterial richness was biomass. This reduction suggests one of the two possible mechanisms: either strong selection within microbiomes to form reduced diversity or emergent dominance of new species (Moreira-Grez et al., 2019). Unlike bacterial richness, an increase in amino acid N decreased fungal richness and an increase in soil inorganic N had no effects on fungal richness; the dominant controls for fungal richness were aboveground biomass and leaf carbon. Our data, combined with previous data (Chen et al., 2019), suggest that the controls for soil bacteria and fungi differ distinctly.

Interestingly, different fungal functional guilds also exhibited contrasting sensitivity to soil N dominance. Specifically, saprotrophic fungi were unchanged with soil N dominance, and both pathogenic fungi and arbuscular mycorrhizal fungi were suppressed by amino acid N dominance but not inorganic N dominance. This suppression could be partly linked to the lack of efficient exoenzymes (*cf.* Rozmoš et al., 2022). Additionally, organic N utilization by arbuscular mycorrhizal fungi can be mediated by specific soil bacteria and protists (Rozmoš et al., 2022). Given that pathogenic fungi and arbuscular mycorrhizal fungi commonly exhibit opposite functioning (Tate, 2021), amino acid N could yield profound consequences for fungi-driven ecological processes.

The use of network analysis allows us to identify theoretical relationships among microbes (Newman, 2003). To date, limited studies have addressed bacteria-fungi networks, although network thinking has long been recognized (Proulx et al., 2005). The Mantel test showed that the dominant control for keystone species was soil pH. In other words, soil pH could shape the co-occurrence networks of soil bacterial and fungal communities by altering interspecific associations (Tate, 2021). While soil pH decreased toward the two ends of the soil N dominance gradient, its role was opposite. Specifically, the inorganic N-induced pH decrease increased the number of keystone species while the amino acid N-induced pH decrease reduced the number of keystone species. Thus, these changes could influence the functioning of microbial communities (Banerjee et al., 2018).

Topological structure determines system stability (Newman, 2003; Hugerth and Anderson, 2017). There were distinct differences in topological properties among the three soil N regimes. These differences suggest that an increase in soil inorganic N or amino acid N could substantially enhance the stability of microbial communities *via* different topological pathways. This pattern might be the result of N selections because an equal proportion of soil inorganic and organic N is relatively rare in the field (Yu and He, 2021). In other words, soil microbes are usually faced with inorganic N dominance or organic N dominance in nature.

Our findings might have some implications. First, soil microbiomes are highly diverse, in part because soil environmental conditions are so heterogeneous (Fierer, 2017). In nature, soil habitats are quite patchy in the relative dominance of soil inorganic and organic N (Yu and He, 2021). Thus, this patchiness could provide new insights into soil microbial patterns. Second, the relative dominance of soil inorganic and organic N appears to be an important factor driving microbial processes. However, this aspect has been ignored in previous studies. Third, there are disparate pathways that mediate soil bacteria and fungi, thereby carefully extrapolating experimental results from given microbial components.

It should be noted that amino acids provide N and C sources for soil microbes simultaneously (Lipson and Näsholm, 2001; Kuzyakov and Xu, 2013; Warren, 2014). Thus, amino acid N sources could have confounding effects. However, similar approaches have been widely employed in previous studies (*cf.* Rozmoš et al., 2022). In our study, we set up the same amount of available N based on the field setting to highlight the effects of inorganic and organic N forms. Undoubtedly, it remains challenging to disentangle the specific effects of N and C sources when adding organic compounds. This problem could stimulate more studies and would be resolved largely due to improvements in technology in the future.

This study provides avenues for further studies. First, there is a need to conduct multi-scale studies in the context of soil N dominance because these studies help us to better understand the composition and co-occurrence networks of soil microbial communities in the field. Furthermore, it is more valuable to explore the patterns of soil microbiomes and their mapping in a realistic setting (Martiny et al., 2006). Second, more attention should be paid to identify the functions of spatiotemporally changing soil microbiomes because functional changes can predict the ecological role of soil microbial communities under environmental change (Hicks et al., 2022).

## Data availability statement

The raw sequencing data have been deposited in the NCBI Sequence Read Archive under accession number: SRP409721.

## Author contributions

W-MH designed and performed the study. YX, YS, and W-MH performed data analysis and drafted the manuscript. All authors contributed to the article and approved the submitted version.

## Funding

This study was funded by the Hebei Agricultural University Talents Fund (YJ2020052), the National Key Research and Development Project (2022YFC2601102), and the Natural Science Foundation of Hebei Province (C2022204058).

## Conflict of interest

The authors declare that the research was conducted in the absence of any commercial or financial relationships that could be construed as a potential conflict of interest.

## Publisher’s note

All claims expressed in this article are solely those of the authors and do not necessarily represent those of their affiliated organizations, or those of the publisher, the editors and the reviewers. Any product that may be evaluated in this article, or claim that may be made by its manufacturer, is not guaranteed or endorsed by the publisher.

## Footnotes

^1^ http://mem.rcees.ac.cn:8081/
