# Supplementary material for: A shift from inorganic to organic nitrogen-dominance shapes soil microbiome composition and co-occurrence networks
Source: Front Microbiol. 2022 Dec 19;13:1074064. doi: 10.3389/fmicb.2022.1074064 (PMC9807163; doi:10.3389/fmicb.2022.1074064)
Supplement: Supplementary file 1 [file Data_Sheet_1.docx]

**A shift from inorganic to organic nitrogen-dominance shapes soil microbiome composition and co-occurrence networks**

**Supporting Information**

**Table S1** Dissimilarity of soil bacterial and fungal communities based on three non-parametric tests.

**Table S2** Relative abundance of phylum-level bacteria and class-level fungi among three soil N regimes.

**Table S3** Taxonomic composition of keystone species in microbial networks.

**Table S1** Dissimilarity of soil bacterial and fungal communities based on three non-parametric tests. INO: inorganic N-dominated; CIO: co-dominated by inorganic and organic N; ORG: organic N-dominated. MRPP: multiple-response permutation procedure; ANOSIM: analysis of similarities; PERMANOVA: permutational multivariate analysis of variance. The values of *P* < 0.05 are in bold.

| Microbe | N regime | MRPP | | ANOSIM | | PERMANOVA | |
| --- | --- | --- | --- | --- | --- | --- | --- |
|  |  | Delta | *P* | R | *P* | F | *P* |
| Bacteria | INO vs. CIO | 0.373 | **0.001** | 0.425 | **0.001** | 2.090 | **0.001** |
|  | INO vs. ORG | 0.436 | **0.001** | 0.985 | **0.001** | 6.765 | **0.001** |
|  | CIO vs. ORG | 0.423 | **0.001** | 0987 | **0.001** | 8.120 | **0.001** |
| Fungi | INO vs. CIO | 0.627 | **0.001** | 0.305 | **0.001** | 1.894 | **0.001** |
|  | INO vs. ORG | 0.635 | **0.004** | 0.295 | **0.003** | 1.895 | **0.001** |
|  | CIO vs. ORG | 0.620 | **0.001** | 0.553 | **0.001** | 2.481 | **0.001** |

**Table S2** Relative abundance of phylum-level bacteria and class-level fungi among three soil N regimes. INO: inorganic N-dominated; CIO: co-dominated by inorganic and organic N; ORG: organic N-dominated. Different lowercase letters indicate significant differences at *P* < 0.05.

|  | Phylum/Class | INO | CIO | ORG |
| --- | --- | --- | --- | --- |
| Bacteria | Proteobacteria | 44.60 ± 1.52 ab | 43.34 ± 1.21 b | 48.94 ± 0.82 a |
|  | Bacteroidota | 14.53 ± 0.54 ab | 16.60 ± 0.63 a | 12.61 ± 0.75 b |
|  | Acidobacteriota | 13.46 ± 0.86 a | 13.58 ± 0.77 a | 9.60 ± 0.75 b |
|  | Actinobacteriota | 5.30 ± 0.19 b | 4.38 ± 0.09 b | 8.10 ± 0.44 a |
|  | Gemmatimonadota | 6.29 ± 0.32 a | 6.15 ± 0.25 a | 3.53 ± 0.13 b |
|  | Myxococcota | 3.44 ± 0.14 ab | 3.39 ± 0.17 b | 4.06 ± 0.14 a |
|  | Verrucomicrobiota | 2.10 ± 0.12 b | 2.49 ± 0.09 ab | 2.86 ± 0.23 a |
|  | Planctomycetota | 1.90 ± 0.21 a | 2.22 ± 0.19 a | 3.14 ± 0.79 a |
|  | Chloroflexi | 2.15 ± 0.14 a | 2.23 ± 0.12 a | 1.97 ± 0.32 a |
|  | Firmicutes | 1.94 ± 0.33 a | 1.06 ± 0.11 b | 1.22 ± 0.08 ab |
|  | Others | 4.30 ± 0.18 ab | 4.65 ± 0.11 a | 3.97 ± 0.13 b |
| Fungi | Sordariomycetes | 32.16 ± 3.81 a | 38.28 ± 4.46 a | 38.62 ± 2.66 a |
|  | Dothideomycetes | 17.30 ± 2.18 a | 18.02 ± 2.70 a | 15.97 ± 0.82 a |
|  | Eurotiomycetes | 8.07 ± 3.61 a | 6.18 ± 1.34 a | 4.17 ± 0.86 a |
|  | Mortierellomycetes | 0.56 ± 0.21 ab | 6.03 ± 4.03 a | 0.10 ± 0.03 b |
|  | Tremellomycetes | 1.61 ± 0.35 ab | 1.03 ± 0.09 b | 3.15 ± 0.8 a |
|  | Leotiomycetes | 2.00 ± 0.70 a | 1.59 ± 0.45 a | 1.09 ± 0.20 a |
|  | Chytridiomycetes | 2.62 ± 1.47 a | 0.84 ± 0.28 a | 0.28 ± 0.09 a |
|  | Agaricomycetes | 1.42 ± 0.86 a | 0.50 ± 0.09 a | 0.78 ± 0.21 a |
|  | Spizellomycetes | 0.46 ± 0.12 ab | 1.00 ± 0.32 a | 0.17 ± 0.05 b |
|  | Blastocladiomycetes | 0.05± 0.02 b | 0.18 ± 0.08 ab | 0.47 ± 0.22 a |
|  | Others | 33.30 ± 6.24 a | 26.35 ± 4.53 a | 35.21 ± 1.86 a |

**Table S3** Taxonomic composition of keystone species in microbial networks. INO: inorganic N-dominated; CIO: co-dominated by inorganic and organic N; ORG: organic N-dominated.

| N regime | ASVID | Category | Kingdom | Phylum | Class |
| --- | --- | --- | --- | --- | --- |
| INO | FunASV25 | Connectors | Fungi | Ascomycota | Sordariomycetes |
|  | FunASV38 | Connectors | Fungi | Ascomycota | Sordariomycetes |
|  | FunASV75 | Connectors | Fungi | Unidentified | Unidentified |
|  | FunASV103 | Connectors | Fungi | Ascomycota | Sordariomycetes |
|  | FunASV156 | Connectors | Fungi | Ascomycota | Leotiomycetes |
|  | BactASV70 | Connectors | Bacteria | Proteobacteria | Alphaproteobacteria |
|  | BactASV106 | Connectors | Bacteria | Proteobacteria | Gammaproteobacteria |
|  | BactASV121 | Connectors | Bacteria | Proteobacteria | Gammaproteobacteria |
|  | BactASV126 | Module hubs | Bacteria | Proteobacteria | Gammaproteobacteria |
|  | BactASV243 | Module hubs | Bacteria | Proteobacteria | Alphaproteobacteria |
| CIO | FunASV12 | Module hubs | Fungi | Ascomycota | Dothideomycetes |
|  | FunASV17 | Connectors | Fungi | Ascomycota | Eurotiomycetes |
|  | FunASV27 | Connectors | Fungi | Unidentified | Unidentified |
|  | FunASV58 | Connectors | Fungi | Ascomycota | Dothideomycetes |
|  | FunASV260 | Connectors | Fungi | Ascomycota | Eurotiomycetes |
|  | BactASV2 | Module hubs | Bacteria | Proteobacteria | Gammaproteobacteria |
|  | BactASV50 | Module hubs | Bacteria | Acidobacteriota | Holophagae |
| ORG | BactASV43 | Connectors | Bacteria | Proteobacteria | Alphaproteobacteria |
|  | BactASV118 | Connectors | Bacteria | Proteobacteria | Gammaproteobacteria |
